# Biostimulatory Effects of Low-Intensity Pulsed Ultrasound on Rate of Orthodontic Tooth Movement and Associated Pain, Applied at 3-Week Intervals: A Split-Mouth Study

**DOI:** 10.1155/2021/6624723

**Published:** 2021-05-05

**Authors:** Irfan Qamruddin, Mohammad Khursheed Alam, Verda Mahroof, Meenaz Karim, Mubassar Fida, Mohd Fadhli Khamis, Adam Husein

**Affiliations:** ^1^Head of Orthodontic Department, Sindh Institute of Oral Health Sciences, Jinnah Sindh Medical University, Karachi, Pakistan; ^2^Orthodontic Department, College of Dentistry, Jouf University, Sakaka 72721, Aljouf, Saudi Arabia; ^3^Orthodontic Department, Baqai Medical University, Karachi, Pakistan; ^4^Prosthodontic Unit, School of Dental Sciences, Universiti Sains Malaysia, Health Campus, 16150 Kubang Kerian, Kota Bharu, Kelantan, Malaysia; ^5^Orthodontics Residency Programme, Section of Dental Surgery, Department of Surgery, Aga Khan University, Karachi, Pakistan; ^6^Oral Biology Unit, School of Dental Science, Universiti Sains Malaysia, Kota Bharu 16150, Kelantan, Malaysia; ^7^Hospital Universiti Sains Malaysia, 16150 Kubang Kerian, Kota Bharu, Kelantan, Malaysia

## Abstract

**Objective:**

Low-intensity pulsed ultrasound (LIPUS) is a noninvasive modality to stimulate bone remodeling (BR) and the healing of hard and soft tissues. This research evaluates the biostimulatory effect of LIPUS on the rate of orthodontic tooth movement (OTM) and associated pain, when applied at 3-week intervals.

**Methods:**

Twenty-two patients (11 males and 11 females; mean age 19.18 ± 2.00 years) having Angle's Class II division 1 malocclusion needing bilateral extractions of maxillary first bicuspids were recruited for this split-mouth randomized clinical trial. After the initial stage of alignment and leveling with contemporary edgewise MBT (McLaughlin–Bennett–Trevisi) prescription brackets (Ortho Organizers, Carlsbad, Calif) of 22 mil, followed by extractions of premolars bilaterally, 6 mm nickel-titanium spring was used to retract the canines separately by applying 150 g force on 0.019 × 0.025-in stainless steel working archwires. LIPUS (1.1 MHz frequency and 30 mW/cm^2^ intensity output) was applied for 20 minutes extraorally and reapplied after 3 weeks for 2 more successive visits over the root of maxillary canine on the experimental side whereas the other side was placebo. A numerical rating scale- (NRS-) based questionnaire was given to the patients on each visit to record their weekly pain experience. Impressions were also made at each visit before the application of LIPUS (T1, T2, and T3). Models were scanned with a CAD/CAM scanner (Planmeca, Helsinki, Finland). Mann–Whitney *U* test was applied for comparison of canine movement and pain intensity between both the groups.

**Results:**

No significant difference in the rate of canine movement was found among the experimental (0.90 mm ± 0.33 mm) and placebo groups (0.81 mm ± 0.32 mm). There was no difference in pain reduction between experimental and placebo groups (*p* > 0.05).

**Conclusion:**

Single-dose application of LIPUS at 3-week intervals is ineffective in stimulating the OTM and reducing associated treatment pain.

## 1. Introduction

As face and smile is the core of communication, people from different walks of life have become more aware of their dentofacial proportions and facial esthetics. More and more people are seeking fixed orthodontic treatment, but their prime concern is the lengthy course of treatment and discomfort associated with tooth movement [1]. Orthodontic tooth movement is a complex process of bone resorption and deposition in response to mechanical force [2], which involves sequential mechanical cyclical stretches of periodontal ligaments, fluid shear stress and compression, inflammatory cytokine production, and cellular differentiation and multiplication, followed by remodeling of the surrounding [3, 4].

Acceleration of bone remodeling under physiological conditions is highly desirable in orthodontic patients to reduce the treatment duration. Several surgical procedures (corticotomies), pulsed electromagnetic fields, direct electrical current, and biomolecule injections may accelerate bone remodeling, but the challenge here is to accelerate bone remodeling in a noninvasive manner [5–7]. Among the least invasive procedures, low-level laser therapy and mechanical vibration have recently gained some popularity in expediting the orthodontic tooth movement and also minimizing the associated pain; however, the results are not predictable [8–11].

In this regard, low-intensity pulsed ultrasound (LIPUS) has been shown to enhance cell metabolism. Its efficacy for bone regeneration and healing of fractures has long been proven for which it is approved by the US Food and Drug Administration and the UK National Institute for Health and Care Excellence [12, 13]. Mechanical loading of bone is pertinent to maintain its mass and strength. When a bone is physiologically loaded, the fluid in the spaces surrounding bone cells produces fluid shear stress that stimulates different cell lines of bone. LIPUS works on the principle of mechanotransduction where external acoustic waves convert fluid shear stress into biochemical changes at a cellular level [14]. In vitro studies have revealed that LIPUS promotes differentiation of bone-forming cells and extracellular matrix formation through modulation of growth factors and other signaling factors [15]. Although very limited research studies have been conducted to assess the effects of LIPUS on orthodontic tooth movement, few animal-based studies have revealed the acceleratory effect of LIPUS on the rate of tooth movement [16, 17]. Low level of toxicity, low immunogenicity, noninvasiveness, and highly targeted approach make it a suitable adjunct to conventional treatment. However, varied techniques, different application strategies, and ultrasound specifications might pose difficulty to clinicians to get the desired results [18].

Pain wearing orthodontic appliances experience varying degrees of pain. Nearly 99% of patients experience some form of discomfort. Patients experience it as soreness and a feeling of compression and stretch in the affected teeth. It results in a decline in oral health (often manifests as weight loss), compromising the masticatory performance and speech. More often they become indifferent to treatment outcomes and stop cooperating [19]. Therefore, it is a matter of concern to find an approach that reduces pain without jeopardizing bone remodeling.

The aim of our research was to evaluate the effectiveness of a single dose of LIPUS on the tooth retraction phase of OTM and the pain associated with it.

## 2. Materials and Methods

This is a randomized clinical trial conducted in the Orthodontic department of Baqai Medical University, Karachi, Pakistan. The study duration was nine months from October 2015 to July 2016. Ethical approval was obtained by the Ethics Committee of Baqai Medical University. Written consent was taken from the patients and the guardians of minors prior to all diagnostic records. The sample size was calculated using power analysis, based on the tooth movement objective. The sample size was determined using power analysis, having 80% power; alpha which indicates significance level was set at 0.05. According to the sample size calculation, twenty-two Pakistani patients, ages ranging from 15 to 30 years (19.18 ± 2.00 years), were selected for the study. Subjects who fell under the following criteria were selected:

1. Male and female subjects with age between 15 and 30 years with a full set of permanent dentition and no missing or impacted teeth except for the third molars
2. No systemic disease or pregnancy
3. Patients having half cusp class II molar relationship, necessitating exclusively bilateral bicuspid extraction
4. Good oral hygiene and compliance

The exclusion criteria include the following:

1. Chronic use of nonsteroidal anti-inflammatory drugs, corticosteroids, and bisphosphonates
2. Patients with any metabolic bone disease
3. Patients with a previous history of fixed orthodontic treatment

### 2.1. Randomization and Study Design

To ensure maximum efficacy, a split-mouth design best suited the study. The right and left sides of the patients who fulfilled the criteria were randomly divided into experimental and placebo groups by a simple randomization technique. Tossing a coin for each patient that enters the trial such that head for the experimental group and tail for the placebo group. Patients did not know which side was experimental or placebo; however, the clinician knew it. The experimental group received the LIPUS irradiation extraorally on the canine root; the transducer was kept at the placebo side for the same duration without turning it on. Blinding was satisfactory as US waves are inaudible and imperceivable.

### 2.2. Methodology

Treatment was initiated with banding and bonding procedures. Preadjusted edgewise MBT prescription brackets (Ortho Organizers, Carlsbad, Calif) of 0.22-in slot were glued following conventional steps of etching and bonding.

For leveling and alignment, a series of NiTi wires were placed, starting from 0.014-in heat-activated nickel-titanium (NiTi) wire followed by 0.016-in NiTi, 0.017 × 0.025-in NiTi, and 0.019 × 0.025-in NiTi upgraded after every 21 days. The final working wire was 0.019 × 0.025 SS. First premolars were extracted on both sides on the 21st day of the final working wire placement. A week after extractions, the canine retraction was commenced. Prior to the beginning of canine retraction, proper leveling and alignment of incisors, bilateral symmetry, and correct angulation of both canines were ensured. The incisors were secured together with 0.010-in steel ligature to prevent inadvertent tooth movement during the retraction phase. A horizontal force was applied by stretching a 6 mm close coil NiTi spring up to 150 g through Orthodontic Dynamometer (Forestadent, Germany) and held with a ligature wire between the power arm of the canine and first molar. Patients were told to meticulously maintain oral hygiene and to inform immediately if spring is severed or displaced. They were also discouraged to take analgesics and also advised to note it down if taken for the severity of pain.

**Figure 1:**
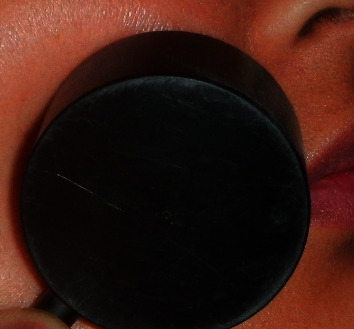
LIPUS application.

Immediately after force application, LIPUS was applied extraorally on the experimental side (Figure 1). Ultrasound gel was applied on the transducer of LIPUS for homogenous penetration, followed by placement over the whole length of the root of the maxillary canine [20]. The transducer of the LIPUS device was also held on the placebo side without turning the device on, so that the placebo design is not disturbed. The procedure was repeated after every 3 weeks after measuring the level of force with the same force measuring gauge, which should be 150 g. Silicone impressions were made before the beginning of retraction (T0) and then were repeated at 3-week intervals for approximately 4 months, i.e., T1, T2, and T3. Dental casts were scanned with Planmeca CAD/CAMTM Lab scanner for the analyses explained in the section later.

### 2.3. LIPUS Specification

LIPUS (Metron accusonic model GS 170 Australia) was used which generates a frequency of 1.1 MHz as it has been used successfully to accelerate BR [21]. The LIPUS wave was delivered in burst for 10 milliseconds followed by a pause of 800 *μ*s. The recommended intensity output for clinical use is 30 mW/cm^2^, which was applied for 20 minutes with a 2.5 cm lead zirconate titanate transducer.

### 2.4. Rate of Canine Retraction

To evaluate the effectiveness of the regimen, the experimental side was compared with the placebo side. A subtle method presented by Gebauer was selected, where *x* and *y* coordinates were drawn on 3D images of the dental cast. Raphe line was taken for the *y*-axis and the medial end of the most prominent rugae was taken for the *x*-axis [22]. The distance between the most distal points on the canine was measured in millimeters from the *x* coordinate in both the groups and measurements on both sides were compared.

### 2.5. Pain Intensity Evaluation

For pain measurement, numerical rating scale was used [8, 9]. The 11-point scale rates the pain intensities with the understanding that 0 stands for no discomfort and 10 for the worst possible pain. Pain recording was commenced four hours after the instigation of spring and patients were asked to record the score that best describes their pain intensity throughout the day after every 24 hours for consecutive 7 days.

### 2.6. Statistical Analysis

The data were recorded, and the results were evaluated on SPSS 20.0 version. Since the data were not normally distributed, nonparametric Mann–Whitney *U* test was used for canine movement and pain comparison.

## 3. Results

Twenty-two patients were selected for the study and the whole process of data collection took seven months. Two patients were dropped out due to spring dislodgement during the retraction, reducing the sample size to twenty patients.

There was no significant difference in canine movement among the two genders. Mann–Whitney *U* test reveals no statistically significant difference in canine movement among experimental and placebo groups. Over a period of 9 weeks, the canine achieved 2.72 mm ± 0.11 movement on the experimental side and 2.45 mm ± 0.98 mm on the placebo side. Moreover, the mean canine movement in experimental groups and placebo groups was 0.90 mm ± 0.33 mm and 0.81 mm ± 0.32 mm, respectively (Table 1).

Our study concludes pain intensity peaked within 24 hours after force activation and subsided at the end of 4th day at most stages of treatment. Females reported a slightly higher score of pain intensity, but the statistical test showed an insignificant difference in pain intensity among the two genders.

**Figure 2:**
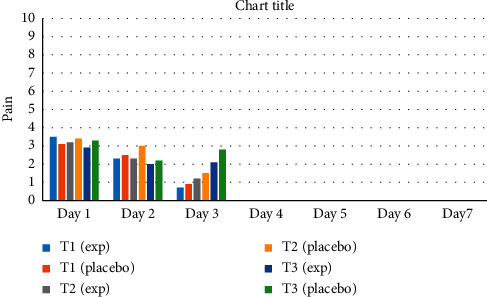
Comparison of pain among the experimental side and placebo side in group A at T1, T2, and T3.

No significant difference was found in the pain intensity between experimental and placebo sides at any stage of treatment (Figure 2).

## 4. Discussion

LIPUS has showed its potent clinical efficacy in soft and hard tissue healing in the field of medicine [23, 24]. Moreover, its effect on the repair and regeneration of orthodontically induced root resorption cannot be overemphasized [20]. It stimulates not only osteogenic cells but also cementoblasts that aid in root regeneration [25, 26]. The effect of LIPUS on OTM and pain in humans has gained little attention.

Our research showed no significant difference in the rate of canine movement among genders as well as among experimental and placebo sides. Since LIPUS has never been tested on humans for its rate accelerating and analgesic effects in orthodontic patients, therefore, direct comparison with similar researches was not possible. However, a marked acceleration in the rate of OTM has been reported in animals [27, 28]. Dahhas applied LIPUS on ovariectomized rats for 28 days at alternate days and found normal orthodontic tooth movement postulating that LIPUS induces normal bone turnover and could be beneficial in orthodontic treatment in postmenopausal women with osteoporosis [27]. Our research investigated a single 20 min application in three weeks suggesting the reason for the ineffectiveness of this treatment regime. On the other hand, Aldagheer applied LIPUS for 20 min for four consecutive weeks on beagle dogs and found no significant acceleration on OTM. Instead, he found that LIPUS diminished resorptive areas on the root by 68% and also reduced the resorption initiation areas by 71% [29]. Few more studies found LIPUS effective in accelerating tooth movement, but the exact biological mechanism has not been completely understood. It has been stipulated from mandibular organ culture study that LIPUS alters tooth movement by promoting alveolar remodeling [30]. Xue in his in vitro rat model study postulated enhanced alveolar bone remodeling through gene expression of HGF/Runx2/BMP-2 signaling pathway. He also applied LIPUS to human PDL cells and observed the expression of BMP-2mRNA and protein due to Runx2 expression which was in agreement with previous research studies [16, 31, 32]. This increased expression of BMP has previously been reported in response to mechanical compression of bone which induces differentiation and proliferation of osteogenic cells inducing bone remodeling [33, 34]. On the contrary, ultrasound also downregulates receptor-activated nuclear factor kappa-B ligand/osteoprotegerin (RANKL/OPG) ratio, tumor necrosis factor-alpha, and interleukin-1b3 which are critical for the differentiation of bone cells and osteoclastic activity [25, 35]. These two contradictory effects of LIPUS may nullify the acceleration and retardation effect of LIPUS on bone remodeling.

LIPUS delivers micromechanical stresses to the tissues. Most of the researchers have applied these micromechanical stresses either on daily basis or on alternate days for at least 28 days to assess the rate accelerating effect of LIPUS on OTM and healing effect on orthodontically induced root resorption [20, 28]. However, we applied a single dose of LIPUS to make it more convenient for the patient, suggesting that this dose is not effective in expediting the rate of orthodontic tooth movement.

Our study did not find any analgesic effect of LIPUS in the reduction of orthodontic pain. The analgesic effect of LIPUS on pain related to OTM has never been investigated previously; however, it has been found efficient in reducing lower back pain and improving the functional ability of patients [36]. Ebadi et al., on the other hand, did not find LIPUS as a modality for analgesia for the management of nonspecific lower back pain [37].

Our clinical trial did not reveal any favorable effect of LIPUS on the rate of OTM and pain. Due to scarce data available in this domain, more studies are required to understand its effectiveness and mechanism of action.

## 5. Conclusion

Single dose of LIPUS applied at 3 weeks neither accelerates the orthodontic tooth movement nor reduces the pain associated with orthodontic tooth movement.

## Figures and Tables

**Table 1:** Mean values and standard deviation of canine movements in experimental and placebo groups with confidence interval and *p* values.

	Experimental side mm (SD)	95% confidence interval		Placebo side mm (SD)	95% confidence interval		Mean difference	*p* value
		Lower bound	Upper bound		Lower bound	Upper bound		
T0-T1	0.97 (0.28)	0.78	1.16	0.79 (0.37)	0.54	1.04	0.18	0.251
T1-T2	0.86 (0.59)	0.46	1.25	0.64 (0.47)	0.32	0.96	0.22	0.253
T2-T3	0.89 (0.31)	0.68	1.11	1.02 (0.14)	0.93	1.12	−0.13	0.433

^*∗*^Significant at*p* < 0.05 (Mann–Whitney *U* test).

## Data Availability

The data used to support the findings of this study are included within the article as Table 1 and Figure 2. Raw data are available from the corresponding author upon reasonable request.
